# Efficacy of cefquinome and a combination of cloxacillin and ampicillin for treatment of dairy cows with *Streptococcus agalactiae* subclinical mastitis

**DOI:** 10.1371/journal.pone.0216091

**Published:** 2019-04-25

**Authors:** Rodolfo Santos Rossi, Ariadne Ferreira Amarante, Simony Trevisan Guerra, Giulia Soares Latosinski, Bruna Fernanda Rossi, Vera Lucia Mores Rall, Jose Carlos de Figueiredo Pantoja

**Affiliations:** 1 Department of Veterinary Hygiene and Public Health, School of Veterinary Medicine and Animal Science, Sao Paulo State University (UNESP), Botucatu, SP, Brazil; 2 Department of Microbiology and Immunology, Institute of Biosciences, Sao Paulo State University (UNESP), Botucatu, SP, Brazil; University of Illinois, UNITED STATES

## Abstract

A randomized clinical trial was conducted to assess efficacy of intramammary cloxacillin and ampicillin (**CLOXIMM**), intramammary cefquinome (**CEFIMM**), and intramuscular cefquinome (**CEFIM**) to treat *Streptococcus agalactiae* intramammary infections (Trial 1). Subsequently, two treatment groups were extended to assess whether CLOXIMM was not inferior to CEFIMM (Trial 2). Nine farms were included in the study. Milk samples were collected from all quarters of all lactating cows for microbiological identification of *S*. *agalactiae*. Positive cows were randomly allocated into four groups: CLOXIMM, CEFIMM, CEFIM, or negative control (**CONTROL**). Study outcomes were bacteriological cure at 14 (**CURE14**), 21 (**CURE21**), and 14 and 21 (**CURE1421**) days after treatment onset, and somatic cell count. Logistic regression was used to estimate the odds of cure between each treatment and CONTROL. Non-inferiority analysis was performed considering a one-sided 95% confidence interval (**CI**) and non-inferiority margins (Δ) of 0.10, 0.15, 0.20, and 0.25. Adjusted *S*. *agalactiae* bacteriological cure for CLOXIMM, CEFIMM, CEFIM, and CONTROL was 86, 98, 55, and 25% at day 14; 82, 93, 52, and 0% at day 21; and 82, 92, 40, and 0% at days 14 and 21, respectively. Treatment with CLOXIMM and CEFIMM resulted in greater bacteriological cure rates, as compared with CEFIM or CONTROL, which does not justify the use of CEFIM in *S*. *agalactiae* eradication programs. The CURE14 difference between CEFIMM and CLOXIMM was of 12.1 percentage points (95% CI: 0.056–0.184). CLOXIMM was considered not inferior to CEFIMM for Δ = 0.20 or 0.25 and inconclusive for Δ = 0.10 or 0.15. Thus, it should be pondered by veterinarians whether an expected 12.1 (5.6–18.4) percentage points increase in cure rate would justify the use of a fourth-generation cephalosporin, as opposed to a combination of traditional IMM drugs (cloxacillin and ampicillin) to treat *S*. *agalactiae* subclinical mastitis.

## Introduction

Control of contagious mastitis caused by *Streptococcus agalactiae* is still challenging in many dairy regions of the world. Reported prevalences at cow level ranged from 7.1% in Thailand [1], 16% in China [2], to 27–35% in Colômbia [3,4]. In Brazil, herd and cow level prevalences have been reported as high as 60% [5], and 21% [6], respectively. Moreover, a reemergence of *S*. *agalactiae* has been observed in countries that had long adopted contagious mastitis control programs. Katholm *et al*. [7] reported that herd prevalence of *S*. *agalactiae* increased from 2 to 6.1% in Denmark between 2000 and 2009.

Intramammary infections (**IMI**) caused by *S*. *agalactiae* result in economic losses to farmers and dairy industries. Infected cows experience milk production losses of 1.6–4.5 Kg/day [8, 9] and can shed up to 10^7^ bacteria/mL [10] and an average of 2,238,000 cells/mL in their milk [11]. Additional economic losses result from recurrent episodes of clinical mastitis experienced by chronically infected cows [1]. *S*. *agalactiae* IMI results in raw milk alterations, such as increased rate of lipolysis and proteolysis [12, 13], which negatively affect its industrial quality, yield, and shelf life.

Historically, implementation of control programs for eradication of contagious mastitis resulted in a drastic decrease in the prevalence of *S*. *agalactiae* in developed dairy regions [14]. A treatment approach named “blitz-therapy” was the basis of such programs and consists of systematic identification and simultaneous treatment of all infected cows [15]. Several researchers have demonstrated that “blitz-therapy” is economically viable when used in *S*. *agalactiae* eradication programs [16–18]. Traditionally, once cows are diagnosed with *S*. *agalactiae* by means of microbiological examination of composite milk samples, all quarters of all infected animals are simultaneously treated with natural or semi-synthetic penicillins, such as cloxacillin [19]. Bacteriological cure rates of 77–100% have been reported following intramammary (**IMM**) treatment with those drugs [4, 18, 20–23].

As an alternative to penicillins, cephalosporins have been used in “blitz-therapy” programs. Cefquinome is a fourth-generation cephalosporin characterized by a broad-spectrum and stability against penicilinases and beta-lactamases [24], available for IMM and intramuscular (**IM**) administration for mastitis treatment. Possible advantages are short treatment duration (1.5 days for IMM use) and milk withdrawal period (60 and 12 hours after the last IMM or IM administration, respectively). Cefquinome is also recommended for mastitis systemic treatment, although little research has demonstrated its ability to reach proper concentrations within the mammary tissue [25]. Systemic treatment of *S*. *agalactiae* subclinical mastitis with cefquinome has been appealing to farmers because treating multiple infected quarters with a single course of IM treatment could be more cost-effective than using several IMM tubes.

Nonetheless, fourth generation cephalosporins should be used with caution in livestock animals to prevent development of resistant bacterial strains. Such antimicrobials are listed by the World Health Organization as “critically important” [26] and its use in farm animals could be avoided if other traditional drugs, such as cloxacillin, are as efficient to treat *S*. *agalactiae* IMI. In this context, this study was divided into two trials, with the following objectives:

Trial 1: to assess the efficacy of IMM cloxacillin (**CLOXIMM**), IMM cefquinome (**CEFIMM)**, and IM cefquinome (**CEFIM)** to treat *S*. *agalactiae* IMI.

Trial 2: to assess whether treatment efficacy of *S*. *agalactiae* IMI with CLOXIMM is not inferior to treatment with CEFIMM.

## Material and methods

This study was approved by the Sao Paulo State University´s Ethics Committee for Animal Use, protocol 07/2015. This article was prepared according to the REFLECT statement for reporting of clinical trials [27].

### Study design

A parallel, non-blinded randomized clinical study was conducted to estimate the efficacy of CLOXIMM, CEFIMM, and CEFIM, as compared with a negative control group (**CONTROL**), to treat *S*. *agalactiae* IMI (Trial 1). Subsequently, two treatment groups (CLOXIMM and CEFIMM) were extended (Trial 2) to achieve the required sample size needed to test the non-inferiority hypothesis proposed in Trial 2´s objective.

### Inclusion and exclusion criteria for herds and cows

Herds were eligible to participate if located in Sao Paulo or Minas Gerais states, Brazil, had bulk tank milk somatic cell count (**SCC**) > 700.000 cells/mL on the last official test day, had *S*. *agalactiae* isolated from bulk tank milk, milked > 20 lactating Holsteins or Holstein crossbred cows, milked cows with milking machines, and offered voluntary cooperation to perform the proposed activities.

Cows were eligible for inclusion if had at least one quarter infected with *S*. *agalactiae*, had a unique identification, were apparently healthy, and had not received any antimicrobial treatment within 15 days prior to inclusion in the study. Cows were excluded from the study if developed any disease after inclusion, died, left the herd for any reason, or received any antimicrobial treatment between the first *S*. *agalactiae* diagnosis (screening day) and beginning of any experimental treatment.

### Sampling strategy

Field representatives of known dairy processors or cooperatives were asked to make a list of herds that attended the inclusion criteria. All farms that attended the criteria and demonstrated interest in participating in the study were included. Initially, four farms were included to accomplish Trial 1´s objective, and five additional farms were included to accomplish Trial 2´s objective. Farms were first visited to explain the study protocol and obtain informed consent. Farms were then revisited on a screening day (**SD**) and aseptic milk samples (15 mL) were collected from all quarters of all lactating cows for microbiological screening of *S*. *agalactiae* IMI. Cows who met the inclusion criteria were then randomly assigned to the study groups.

### Randomization

For Trial 1, cows with at least one quarter infected with *S*. *agalactiae* were randomly allocated into one of four groups, such that all infected quarters within a cow received the same treatment protocol. Within each participant herd, eligible cows were stratified by parity (1 or > 1 lactation) and within each parity stratum, blocked randomization [28] was used to allocate cows into the study groups. Blocks of seven animals were formed and within each block, two cows were randomly allocated into each of the treated groups (CLOXIMM, CEFIMM, or CEFIM), and one cow was allocated into CONTROL. The CONTROL group had fewer cows than the treated groups to minimize the risk of *S*. *agalactiae* transmission between quarters and cows during the course of the study.

For Trial 2, five additional herds were included to extend two treatment groups (CLOXIMM and CEFIMM) and reach the required sample size. Except for the block size (two cows), cows were allocated into CLOXIMM or CEFIMM, following the randomization procedure previously described. Although a smaller sample size was required for Trial 1, data from the nine farms were used in the analysis.

### Treatments and follow-up sampling

Treatment of *S*. *agalactiae* infected quarters was administered upon completion of microbiological diagnosis, attempting not to exceed seven days from SD. Cows in CLOXIMM (N = 94 quarters of 48 cows) received an IMM injector containing 250 mg of cloxacillin and 125 mg of ampicillin (Intramast, Vallée, Sao Paulo, Brazil) every 24 hours, for three days. Cows in CEFIMM (N = 100 quarters of 47 cows) received an IMM injector containing 75 mg of cefquinome (Cobactan VL, MSD Animal Health, Sao Paulo, Brazil) every 12 hours, for 1.5 days (three consecutive milkings). Cows in CEFIM (N = 31 quarters of 12 cows) received an IM injection of cefquinome (1 mg/kg) (MSD Animal Health, Sao Paulo, Brazil) every 24 hours, for three days. Cows in the control group did not receive any treatment or placebo. Milk of treated animals was discarded following label directions, for 72, 60, and 12 hours after the last treatment with CLOXIMM, CEFIMM, and CEFIM, respectively.

Treatments were initially performed by the authors (first milking) and continued until completion by trained farm personnel. After milking of each animal, teat ends were scrubbed with a cotton pad moistened with 70% isopropanol. After IMM infusion, teats and quarters were massaged in an ascending direction to improve the distribution of the drug within the mammary gland. Intramuscular injections were performed in the caudal thigh muscles (semimembranosus or semitendinosus), after antisepsis of the skin.

Duplicate aseptic milk samples were collected by study personnel from all enrolled quarters at 14 ± 2 (**D14**) and 21 ± 2 (**D21**) days after treatment onset, for microbiological examination and SCC. For CONTROL, samples were collected on the same days. Production data, such as parity, days in milk (**DIM**), and milk production were recorded.

Farmers and milking technicians were trained to improve their milking routines and prevent transmission of *S*. *agalactiae* during the study. Training was based on the National Mastitis Council´s (**NMC**) five-point mastitis control plan [29]. Main recommendations were segregation of infected cows in a separate milking group (milked always last), wearing of gloves, use of validated pre- and post-dipping solutions, and drying of teats with disposable paper towels.

### Microbiological examination of milk and somatic cell count

Milk samples were kept refrigerated in ice coolers and processed at Sao Paulo State University´s Mastitis Research and Diagnosis Laboratory, within 24 hours after collection. Milk samples were examined according to the NMC´s procedures [30]. Ten μL of milk were streaked onto a quadrant of a blood agar plate (trypticase soy agar with 5% sheep blood; bioMérieux, Sao Paulo, Brazil), and incubated at 36°C. Samples were read at 24, 48 and 72 hours. An IMI was defined as the presence of pure growth of ≥ 3 similar colonies on the plate. Samples were considered negative (non-significant growth) when there were ≤ 2 similar colonies on the plate, and contaminated when there were ≥ 3 types of colonies on the plate.

When one of the duplicate milk samples collected on D14 or D21was contaminated, the other sample was used for analysis. When one of the duplicate samples was positive for *S*. *agalactiae* and the other was negative, an IMI was confirmed based on the positive result.

Diagnosis of *S*.*agalactiae* was performed based on phenotypic identification of colonies, Gram staining and further biochemical tests. Positive samples were Gram-positive cocci, catalase-negative, aesculin and bile aesculin-negative, and Christie, Atkins, Munch-Petersen test-positive. All isolates were submitted to Polymerase Chain Reaction, as described by Chen *et al*. [31] to confirm the diagnosis.

Somaticell (IDEXX, Sao Paulo, Brazil) was used to perform SCC in quarter milk samples collected on SD, D14, and D21, following the manufacturer´s instructions. Two mL of reagent and milk were mixed in a plastic tube and homogenized for 30 s. The tube was then held upside down for 30 seconds for draining of the solution, and the reading (cells/mL) was performed using the scale on the tube´s wall.

### Study outcomes

For Trial 1, the primary outcome was bacteriological cure, defined as the isolation of *S*. *agalactiae* at SD, followed by a negative culture result (or the isolation of a different pathogen) at D14 (**CURE14**). Secondary outcomes were bacteriological cure at D21(**CURE21**), and at D14 and D21 (**CURE1421**). Another secondary outcome was SCC at D14 and D21. For Trial 2, the outcome was CURE14.

### Sample size calculations

For Trial 1, sample size calculation was performed to detect a predefined difference between proportions and demonstrate that the proportion of bacteriological cure for each treatment was greater than that of CONTROL. Calculations were performed assuming α = 0.05, statistical power = 0.8, expected proportion of cure after IMM or IM therapy = 0.8, and 0.2 for CONTROL [4, 22, 23]. Based on these assumptions, at least 10 quarters per group were required. An adjustment was made to the sample size to consider the smaller size of CONTROL [32]. The calculation was performed considering a ratio of 2:1 between treated and control quarters, maintaining the same statistical power of 0.8. In the final estimate, at least 30 treated quarters per group and 15 control quarters were required to accomplish the analytical objective.

For Trial 2 (non-inferiority test), sample size was determined based on a 95% confidence interval (**CI**) for the bacteriological cure difference between CLOXIMM and CEFIMM, in relation to the non-inferiority margin (Δ) and the null effect margin (difference between treatments = 0) [33]. According to the Consolidated Standards of Reporting Trials Statement [33], Δ is customarily chosen based on traditional clinical trials (with a negative control group), so that the non-inferiority margin is not greater than half of the expected effect of the treated group. Based on a pilot study performed by the authors [34], the maximum non-inferiority margin adopted could be of 0.35 (*S*. *agalactiae* bacteriological cure risk = 0.20 and 0.90 for negative control and treated group, respectively). Although justified by the guidelines aforementioned [33], Δ = 0.35 might be considered too great of a biological difference to consider non-inferiority. Thus, we estimated the sample size considering Δ ranging from 0.15 [35, 36] to 0.20 [37], α = 0.05, statistical power = 0.80, and proportion of bacteriological cure for both CLOXIMM and CEFIMM = 0.90 [34].

Based on these assumptions, 50 quarters per group were needed for the study. Because multiple quarters per cow could be included in the study, an adjustment [38] was applied to account for the clustering of quarters within cow. Assuming an intraclass correlation of 0.28 between quarters infected with *S*. *agalactiae* within the same cow [39], 92 quarters per group were needed to accomplish objective 2.

### Statistical analysis

#### Definitions

When milk samples could not be collected on D14 or D21, quarters were excluded from the calculations of CURE14 and CURE21, respectively. When treated quarters exhibited clinical mastitis before D14, or between D14 and D21, and were treated by milking technicians, a treatment failure was considered [36, 40].

#### Analytical procedures

For Trial 1, the analysis was performed at quarter level. Generalized linear models using a logit link were constructed using PROC GLIMMIX of SAS [41] to estimate the chances of bacteriological cure between each treatment and CONTROL. Separate models were constructed for CURE14, CURE21, and CURE1421. Farm was considered a random effect. Parity, milk yield at treatment, DIM, and number of *S*. *agalactiae* infected quarters per cow were included in the model as covariates and remained if significant, according to a backward variable selection procedure. Days in milk was categorized into 0–140, 141–230, and > 230 DIM, and milk yield was categorized into 0–15, 15–22, and > 22 kg/cow/day, based on the 33^rd^ and 66^th^ percentiles of their distributions. Parity was categorized into 1 and > 1. A compound symmetry covariance structure was used to model the correlation among quarters within the same cow.

For the analysis of CURE21, and CURE1421, the multivariable models did not converge because the data were sparse (no cured quarters in CONTROL). Thus, CONTROL was excluded from these analyses so that statistical comparisons could be made between the treated groups.

A repeated measures mixed model was constructed using PROC MIXED of SAS [41] to compare post-treatment mean SCC (log10 cells/mL) among the study groups. Farm was considered a random effect. Parity, milk yield at treatment, DIM and number of *S*. *agalactiae* infected quarters within cow were included in the model as covariates, and remained if significant, according to a backward variable selection procedure. Post-treatment SCC was excluded from this analysis when a quarter was retreated by milking technicians with any other treatment than those in the study protocol.

For Trial 2, assessment of non-inferiority was performed using adjusted CURE14 estimates derived from the generalized linear models aforementioned. The 95% CI for the adjusted cure risk difference between CEFIMM and CLOXIMM was calculated [33] considering Δ of 0.10, 0.15, 0.20, and 0.25. All analyses were performed at a significance level of 0.05.

## Results

### Herd characteristics

The study was conducted in nine commercial farms located in the states of Sao Paulo and Minas Gerais, between February 2016 and May 2017. On six farms (A, B, C, G, H and I) cows were milked in pit parlors and on three farms (D, E, and F) cows were milked in stanchion parlors. Herd size ranged from 22–82 lactating cows, herds’ average milk yield was between 9.0 and 22.7 kg/cow/day, and bulk tank SCC ranged from 760,000 to 1,900,000 cells/mL (Table 1). All cows were milked twice a day and were housed in semi-confinement systems. At SD, prevalence of cows and quarters infected with *S*. *agalactiae* ranged from 15.5–45.1% and 4.6–25.2% across the herds studied, respectively (Table 1).

**Table 1:** Characteristics of the nine herds enrolled into the study.

	N	Min	Q1^4^	Median	Q3^4^	Max
Bulk tank SCC (x 1,000 cells/mL)^1^	9	760.0	921.0	1,317.0	1,700.0	1,900.0
Number of lactating cows	9	22.0	28.0	40.0	74.0	82.0
Average milk yield (Kg/cow/day)^2^	8	9.0	13.9	18.9	21.4	22.7
Prevalence of *Streptococcus agalactiae* (cow level)^3^	9	15.5	17.9	21.2	37.9	45.1
Prevalence of *Streptococcus agalactiae* (quarter level)	9	4.6	7.8	11.0	21.9	25.2

^1^ Last bulk milk somatic cell count before beginning of treatments.

^2^ Herd average milk yield at treatment onset. Milk yield data from one herd could not be used due to lack of measurement precision.

^3^ A cow was considered positive if at least one quarter was infected with *S*. *agalactiae*, as diagnosed by microbiological examination of quarter milk samples.

^4^ Q1 = 1^st^ quartile; Q3 = 3^rd^ quartile.

### Completeness of the dataset

**Fig 1:**
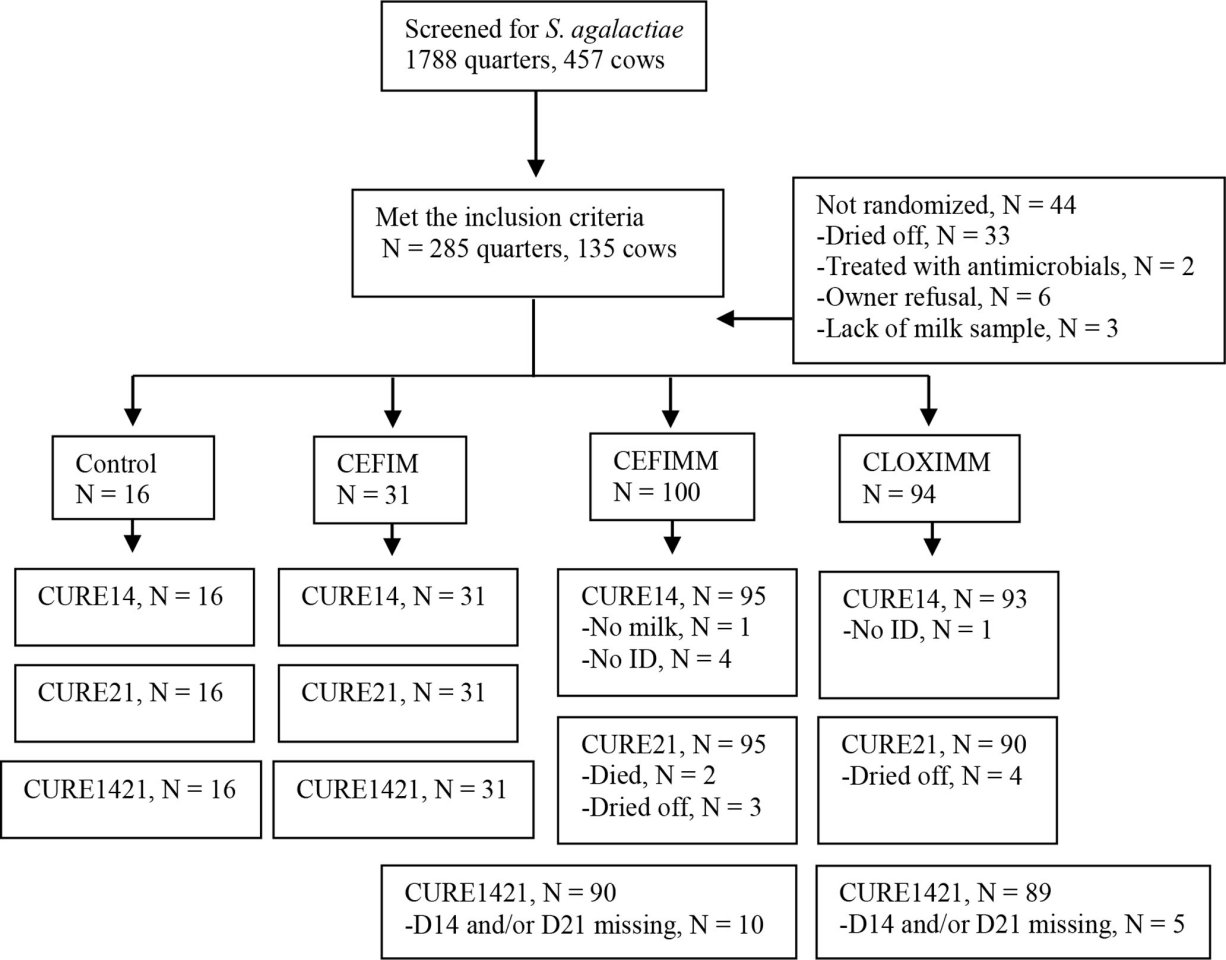
Completeness of the dataset used for analysis. CLOXIMM: quarters treated with an intramammary infusion containing 250 mg of cloxacillin and 125 mg of ampicillin, every 24 hours, for three days. CEFIMM: quarters treated with an intramammary infusion containing 75 mg of cefquinome, every 12 hours, for 1.5 days. CEFIM: quarters treated with intramuscular injection of cefquinome (1 mg/kg) every 24 hours, for three days. Control: quarters did not receive any treatment or placebo. CURE14, CURE21, and CURE1421: bacteriological cure, defined as the isolation of *Streptococcus agalactiae* before treatment, followed by a negative culture result (or the isolation of a different pathogen) at 14, 21, and 14 and 21 days after treatment onset, respectively.

The completeness of the dataset used for analysis is presented in Fig 1. A total of 1,788 quarters of 457 cows were initially screened for *S*. *agalactiae* at SD. Of these, 285 quarters of 135 cows were positive for *S*. *agalactiae* and met the inclusion criteria. Forty-four quarters of 18 cows were not randomized after inclusion, due to the following reasons: two quarters of the same cow were excluded due to antibiotic treatment between SD and initial treatments (herd B). Ten quarters of three cows in herd G and 23 quarters of nine cows in herd H were dried off after SD, and three quarters of one cow in herd A were excluded due to lack of milk on D14 and D21. Six quarters of four cows in herd G were excluded because the owner refused to allow inclusion in the study (cows were knowingly infected with *Staphylococus aureus*).

Six quarters of four cows were excluded from only CURE14 analysis, of which one was due to lack of milk on D14 and five due to lost ear tags on D14. Nine quarters of five cows were excluded from only CURE21 analysis, of which two were excluded due to death between D14 and D21, and seven were dried off.

One quarter of a cow and three quarters of two cows exhibited clinical mastitis prior to D14 and between D14 and D21, respectively, and were treated by milking technicians. Of these four quarters, one was considered a treatment failure for CURE14, CURE21 and CURE1421, and three were considered a treatment failure only for CURE21 and CURE1421.

### Group characteristics and sampling intervals

Cow characteristics (parity, DIM, milk yield, and number of infected quarters per cow) after randomization into the study groups are presented in Table 2. The median interval between SD and initial administration of treatments was seven days. The median interval between initial administration of treatments and the follow-up sampling days (D14 and D21) was 15 and 21 days, respectively.

**Table 2:** Cow characteristics after randomization.

Cow characteristics^2^	Study Group^1^								
	CLOXIMM		CEFIMM		CEFIM		CONTROL		
	Med^3^	(Q1-Q3)^4^	Med	(Q1-Q3)	Med	(Q1-Q3)	Med	(Q1-Q3)	P^5^
Parity	3	(2–4)	2	(1–3)	3	(2–5)	3	(2–5)	0.43
Days in milk at treatment	197	(128–242)	180	(120–265)	200	(94–235)	145	(44–201)	0.52
Milk yield at treatment (Kg/cow/day)	18	(13–27)	22	(16–26)	18	(13–18)	14	(7–24)	0.53
Number of *Streptococcus agalactiae* infected quarters per cow	2	(1–3)	2	(1–3)	3	(2–4)	2	(1–2)	0.06

^1^ CLOXIMM: IMM infusion of cloxacillin (250 mg) and ampicillin (125 mg), every 24 hours, for three days. CEFIMM: IMM infusion of cefquinome (75 mg) every 12 hours, for 1.5 days. CEFIM: IM injection of cefquinome (1 mg/kg), every 24 hours, for three days. CONTROL: did not receive any treatment or placebo.

^2^ Statistics are presented at cow level.

^3^ Median.

^4^ Q1 = 1^st^ quartile; Q3 = 3^rd^ quartile.

^5^ P-value, Kruskal-Wallis test.

### Trial 1

#### Bacteriological cure

Overall unadjusted *S*. *agalactiae* bacteriological cure risk for CLOXIMM, CEFIMM, CEFIM, and CONTROL was 86.02, 97.89, 54.84, and 25.00% at D14; 82.22, 92.63, 51.61, and 0% at D21; and 83.15, 92.22, 51.61, and 0% at D1421, respectively (Table 3).

**Table 3:** *Streptococcus agalactiae* unadjusted bacteriological cure risk.

Bacteriological cure^4^	Study group^1^							
	CLOXIMM		CEFIMM		CEFIM		CONTROL	
	%^2^	N^3^	%	N	%	N	%	N
**CURE14**	86.02	80/93	97.89	93/95	54.84	17/31	25.00	4/16
**CURE21**	82.22	74/90	92.63	88/95	51.61	16/31	0.00	0/16
**CURE1421**	83.15	74/89	92.22	83/90	51.61	16/31	0.00	0/16

^1^ CLOXIMM: IMM infusion of cloxacillin (250 mg) and ampicillin (125 mg), every 24 hours, for three days. CEFIMM: IMM infusion of cefquinome (75 mg) every 12 hours, for 1.5 days. CEFIM: IM injection of cefquinome (1 mg/kg), every 24 hours, for three days. CONTROL: did not receive any treatment or placebo.

^2^ Percentage of cured quarters.

^3^ Number of cured quarters divided by the total number of quarters per group.

^4^ Defined as isolation of *S*. *agalactiae* at herd screening, followed by a negative culture result (or the isolation of a different pathogen) at 14 (CURE14), 21(CURE21), and 14 and 21 days (CURE1421) after treatment onset.

Adjusted *S*. *agalactiae* bacteriological cure risk for each study group are presented in Table 4. Except for study group, none of the covariates included in the multivariable model were significantly associated with the odds of CURE14 (*P* > 0.05). The odds of CURE14 after treatment with CEFIMM (OR = 45.45; 95% CI = 8.70–250.00) or CLOXIMM (OR = 5.85; 95% CI = 2.08–16.39) were significantly higher, as compared with CEFIM.

**Table 4:** Adjusted *Streptococcus agalactiae* bacteriological cure risk.

Variable	Coefficient	SE	*P*-Value	OR^1^	95% CI^2^	Adjusted cure risk^3^ (%)	95% CI^4^
**CURE14**^5^							
**Intercept**	-1.16	0.61					
**Study group**^6^			< 0.01				
CLOXIMM	2.96	0.68		19.67	5.04–73.68	85.77	75.46–92.20
CEFIMM	5.03	0.96		153.30	23.32–999.99^8^	97.96	91.58–99.53
CEFIM	1.19	0.69		3.29	0.86–12.78	50.80	30.67–70.58
CONTROL	Reference			Reference		23.84	8.63–50.92
**CURE21**^5^							
**Intercept**	0.28	0.74					
**Study group**^6^			< 0.01				
CLOXIMM	1.68	0.62		5.38	1.56–18.59	80.99	63.25–91.34
CEFIMM	2.67	0.70		14.50	3.66–57.48	91.99	79.63–97.12
CEFIM	Reference			Reference		44.20	19.14–72.61
CONTROL^7^	-	-		-	-	-	-
**Number of *Streptococcus agalactiae* infected quarters per cow**			0.01				
1	-0.58	0.71		0.56	0.14–2.27	75.87	50.87–90.52
2	0.18	0.75		1.20	0.27–5.26	87.11	66.71–95.79
3	-1.63	0.63		0.20	0.06–0.67	52.30	29.05–74.59
4	Reference			Reference		84.93	61.95–95.13
**CURE1421**^5^							
**Intercept**	-0.04	0.77					
**Study group**^6^			< 0.01				
CLOXIMM	1.94	0.65		6.98	1.93–25.31	82.36	63.58–92.59
CEFIMM	2.84	0.72		17.13	4.16–70.51	91.98	78.69–97.27
CEFIM	Reference			Reference		40.08	15.51–70.92
CONTROL^7^	-	-		-	-	-	-
**Number of *Streptococcus agalactiae* infected quarters per cow**			< 0.01				
1	-0.18	0.72		0.84	0.20–3.48	79.89	54.08–93.06
2	0.34	0.74		1.41	0.33–6.01	87.02	65.50–95.95
3	-1.63	0.61		0.20	0.06–0.65	48.23	24.43–72.87
4	Reference			Reference		82.63	57.18–94.42

^1^ Odds ratio derived from generalized linear models (logistic regression).

^2^ 95% confidence interval for the odds ratio.

^3^ Least square means derived from a generalized linear model (logistic regression).

^4^ 95% confidence interval for the least square mean.

^5^ Defined as isolation of *S*. *agalactiae* at herd screening, followed by a negative culture result (or the isolation of a different pathogen) at 14 (CURE14), 21(CURE21), and 14 and 21 days (CURE1421) after treatment onset.

^6^ CLOXIMM: IMM infusion of cloxacillin (250 mg) and ampicillin (125 mg), every 24 hours, for three days. CEFIMM: IMM infusion of cefquinome (75 mg) every 12 hours, for 1.5 days. CEFIM: IM injection of cefquinome (1 mg/kg), every 24 hours, for three days. CONTROL: did not receive any treatment or placebo.

^7^ Adjusted least square means for CURE21 and CURE1421 were not estimated because the statistical model did not converge (no cure in CONTROL).

^8^ Inestimable upper limit due to data sparseness (few treatment failures).

For CURE21 and CURE1421, the variables treatment and number of *S*. *agalactiae* infected quarters per cow remained in the final model (*P* ≤ 0.01; Table 4).

### Somatic cell count

**Fig 2:**
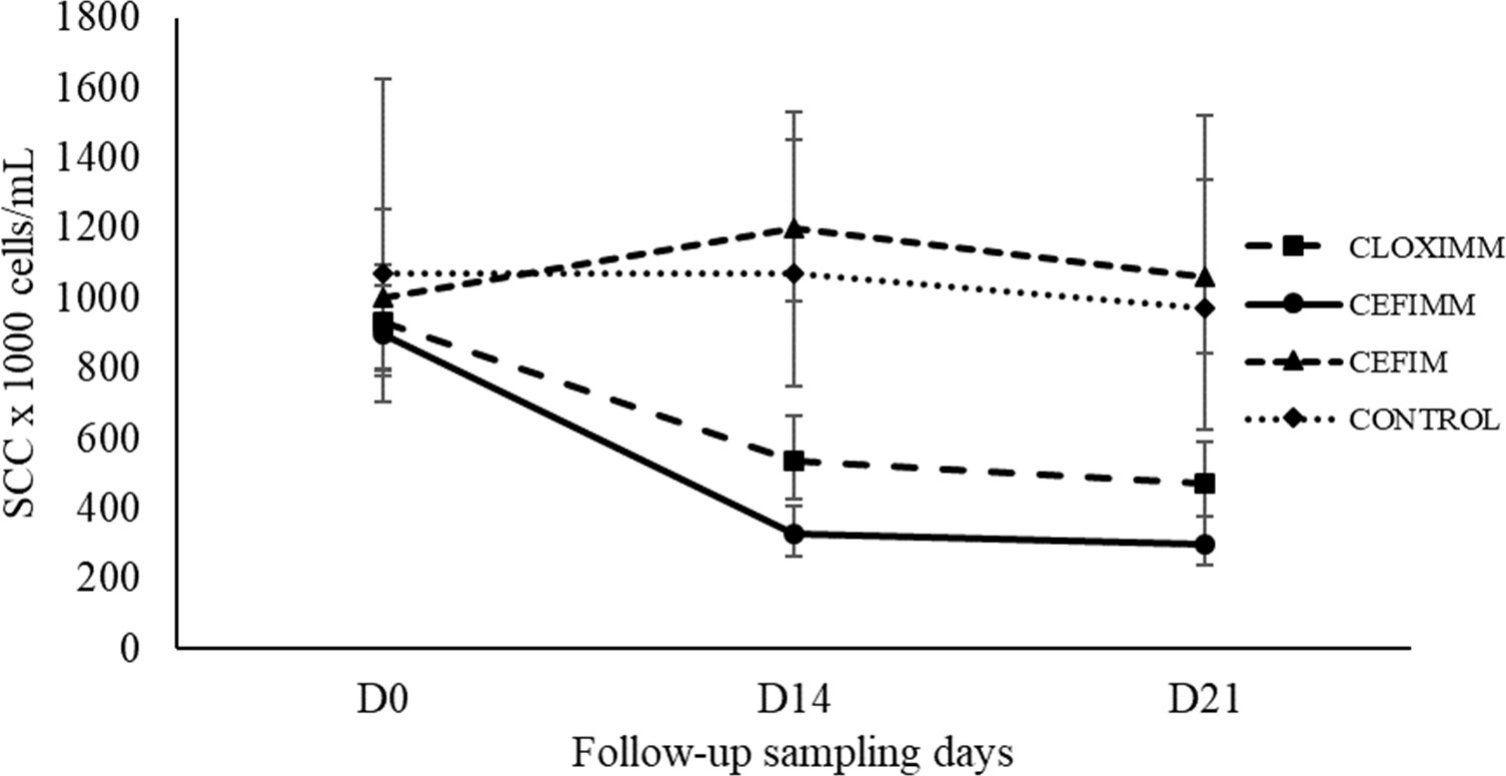
Geometric mean somatic cell count (SCC) of milk samples collected at initial screening (SD), 14 (D14), and 21 (D21) days after treatment onset. Error bars indicate 95% CI of the mean. CLOXIMM: IMM infusion of cloxacillin (250 mg) and ampicillin (125 mg), every 24 hours, for three days. CEFIMM: IMM infusion of cefquinome (75 mg) every 12 hours, for 1.5 days. CEFIM: IM injection of cefquinome (1 mg/kg), every 24 hours, for three days. CONTROL: did not receive any treatment or placebo.

Post-treatment SCC response of quarters treated with CEFIM was not different than that of CONTROL (Fig 2). In contrast, geometric mean SCC decreased 43 and 49% (CLOXIMM) and 64 and 67% (CEFIMM) on D14 and D21, respectively (*P* < 0.01). Mean SCC was different between CLOXIMM and CEFIMM at D14 (*P* < 0.03), but not at D21 (*P* > 0.05; Fig 2).

### Trial 2

#### Bacteriological cure

**Fig 3:**
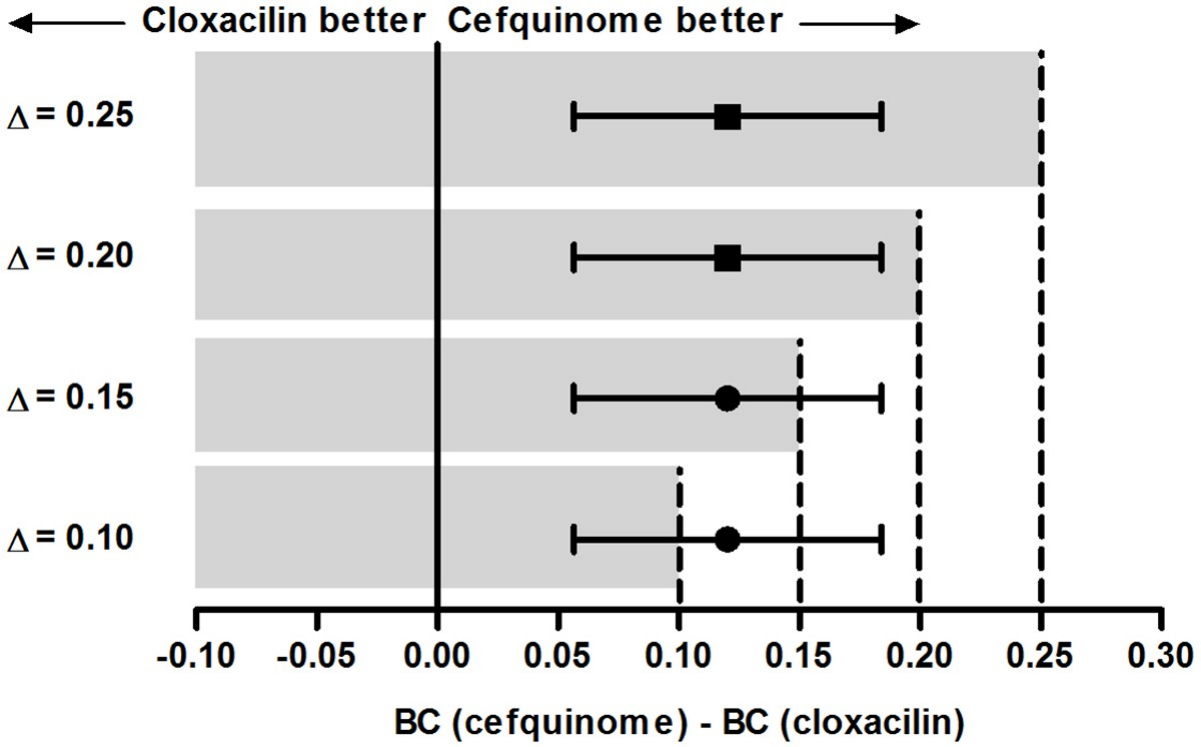
Graphical presentation for possible interpretations of the non-inferiority test considering different non-inferiority margins. Error bars indicate 95% confidence intervals (95% CI) for the difference between bacteriological cure risks. CLOXIMM: IMM infusion of cloxacillin (250 mg) and ampicillin (125 mg), every 24 hours, for three days. CEFIMM: IMM infusion of cefquinome (75 mg) every 12 hours, for 1.5 days. The solid vertical line depicts the null difference between treatments and the dotted vertical line depicts the maximum acceptable difference (0.10, 0.15, 0.20, and 0.25) between treatments to conclude non-inferiority. The grey area depicts the non-inferiority zone for each margin. The horizontal lines with a central square depict the 95% CI in which non-inferiority can be determined. The horizontal line with a central circle depicts the 95% CI in which non-inferiority is inconclusive (Adapted from Piaggio *et al*. [33]).

Adjusted CURE14 was 98.0% and 85.9% for quarters treated with CEFIMM and CLOXIMM, respectively, resulting in a difference of 12.1 percentage points (Δ = 0.121; 95% CI: 0.056–0.184; Fig 3). Treatment with CLOXIMM was considered non-inferior to CEFIMM when Δ of 0.20 and 0.25 were considered, as the upper 95% CI of the difference did not extend beyond the proposed non-inferiority margins. Determination of non-inferiority was inconclusive for Δ of 0.10 and 0.15 since the CI for Δ overlapped both non-inferiority and inferiority areas [33].

## Discussion

Pressure to reduce antimicrobial use in livestock animals has increased in the last decades. Fourth-generation cephalosporins have received special attention due to its classification as critically important for human use and high probability of gene resistance transmission between microorganisms [26, 42]. According to the European Medicines Agency [42], this group should only be used in livestock animals when there is no alternative antimicrobial commercially available.

Results of this study demonstrated that both cefquinome (adjusted cure risk = 98%) and a combination of cloxacillin and ampicillin (adjusted cure risk = 86%) are effective to treat *S*. *agalactiae* IMI when administered intramammarily. *S*. *agalactiae* cure risk after treatment with CLOXIMM agrees with previous reports (98%, [43]; 100%, [20]; 92%, [21]; 77%, [23]; and 82%, [4]). To our knowledge, no studies have been previously conducted to investigate efficacy of IMM cefquinome to treat *S*. *agalactiae*.

The adjusted cure risk difference between CEFIMM and CLOXIMM at D14 (Δ = 0.121; 95% CI: 0.056–0.184) was within the hypothesis that treatment with CLOXIMM is not inferior to CEFIMM considering Δ = 0.20 or 0.25. Nevertheless, the 95% CI of the difference fell outside the limits to conclude non-inferiority when Δ of 0.10 and 0.15 were considered. The choice of Δ in non-inferiority trials is arbitrary [44]. Piaggio *et al*. [33] reported that Δ has been chosen based on clinical trials that use a negative control group, so that Δ is not greater than half of the expected effect of the treated group. Some non-inferiority human clinical trials have aimed to preserve at least 50–70% of the therapeutic effect of the active control [44]. Non-inferiority margins of 0.15 [35, 36] and 0.20 [37] have been used in non-inferiority trials in our field of research. We chose to present different Δ because readers can interpret non-inferiority based on their own biological criteria. Rather than deciding a fixed Δ, the 95% CI for Δ is more important for those who will apply results of this study in the field. Readers should consider that, if this study was consecutively repeated, 95% of the times the CLOXIMM cure risk at D14 would be 5.6–18.4 percentage points lower than that of CEFIMM.

In “blitz -therapy” programs, it should be considered that, out of 10 CLOXIMM treated quarters, 1–2 would remain uncured. While such quarters could act as reservoirs of infection, bacteriological cure would be assessed within seven days after treatment, allowing these infected quarters to be quickly identified and managed accordingly (e.g., retreated). Thus, according to our point estimates, it should be pondered by veterinarians whether a lower cure rate using older IMM drugs (cloxacillin and ampicillin) would be acceptable in “blitz -therapy” programs, as opposed to increasing the cure rate up to 12.1 (5.6–18.4) percentage points, at the cost of using a fourth-generation cephalosporin.

Quarters treated with CEFIM experienced the lowest cure rate (55%) at D14, as compared with CEFIMM or CLOXIMM. Use of IM therapy to treat *S*. *agalactiae* IMI has been appealing for cases in which multiple quarters of the same cow are affected, so that a single course of treatment would treat all quarters at the same time. However, the low CEFIM cure rate observed here suggests that its use cannot be justified in “blitz -therapy” programs for eradication of *S*. *agalactiae*. The low CEFIM cure rate could have been attributed to insufficient penetration of cefquinome into the mammary gland. Cefquinome has a low PKa (2.5–2.9) and low liposolubility, which results in limited penetration into the mammary gland [24]. In contrast, Ehinger *et al*. [25] suggested that cefquinome can penetrate into the mammary tissue, but the study was performed “in vitro”, after simulation of systemic administration in fresh udders removed from slaughtered lactating cows.

It could be hypothesized that the low CEFIM cure rate could have been attributed to reinfections from uncured quarters within the same cow. However, our data show that reinfections did not bias the results because all possible reinfections (11 quarters that were culture-negative on D14 and positive on D21) were distributed across all study groups (five in CEFIMM, two in CONTROL, one in CEFIM, and three in CLOXIMM). Besides, lower reinfection rates within the same cow would have been expected in CEFIM quarters because possible false-negative quarters at SD would have been treated with IM injection.

Interestingly, the odds of CURE21 were lower (OR = 0.20; 95% CI: 0.06–0.67) for cows infected with three quarters than for cows infected with four quarters (Table 4). It is possible that, for cows with three infected quarters, new infections originating from one of these quarters occurred between milk sampling at SD and treatment. These infected quarters were probably left untreated and potentially transmitted *S*. *agalactiae* to the other treated and cured quarters, decreasing the odds of bacteriological cure for these cows.

Bacteriological cure has been traditionally defined as the presence of negative culture results on two successive post-treatment sampling days (e.g., 14 and 21 days) [45, 46]. Because there was a negative control group being managed in the herd, bacteriological cure at 14 days after treatment onset was considered the primary study outcome, to minimize the risk of reinfection by *S*. *agalactiae* between days D14 and D21. The occurrence of reinfections from negative control quarters, or uncured quarters within the same mammary gland could have resulted in underestimation of the treated groups´ actual cure risk. As previously mentioned, only 11 quarters (4.5%) were negative for *S*. *agalactiae* at D14 and positive at D21, suggesting few reinfections between D14 and D21.

The definition of IMI used in this study (300 colony-forming units/mL; [30]) could have resulted in some misclassification of *S*. *agalactiae* IMI. It is possible that infected quarters whose milk had a low concentration of bacteria could have left untreated. It is also possible that *S*. *agalactiae* IMI that had ≥ 3 colonies on D14 and < 3 colonies on the plate at D21 would be misclassified as cured IMI. However, we believe that such misclassification was unlikely to occur because shedding of *S*. *agalactiae* in milk is very high [11]. It is likely that misclassification of IMI did not result in bias because the definition was the same for all study groups.

Milk samples for microbiological examination were not collected at treatment onset because we assumed that spontaneous cure was not likely to occur in such short interval (seven days). If spontaneous cures did occur, it is possible that some quarters were unnecessarily treated, and cure rates could have been overestimated. Nonetheless, selection bias was not likely to have been introduced because the same sampling strategy was used for all groups.

We believe that lack of blinding was not an issue in this study because we randomized cows and initiated treatments ourselves. If treatments were performed by farm personnel, the study could have been more susceptible to selection bias, such as that resulting from purposely selecting better cows (e.g., higher milk production) for inclusion in the treated groups.

Post-treatment SCC response of quarters treated with CEFIM was similar to that of CONTROL quarters, reflecting the low CEFIM cure rate (55%) and the high shedding (average of 2,2 x10^6^ cells/mL; [11]) of somatic cells in milk of infected quarters. Post-treatment SCC response at D21 was not different between CLOXIMM and CEFIMM, and agrees with their respective cure rates. Despite the high cure rates observed in CLOXIMM and CEFIMM, SCC did not return to normal levels (< 200,000 cells/mL) at D21. This could be a result of few uncured quarters that remained in each of these groups, new IMI caused by other pathogens or, most likely, a longer SCC recovery time expected for quarters that were chronically infected before treatment.

The authors believe that results of this study can be extrapolated to a larger population of similar herds and cows as those included in the study. Our findings can be used to improve *S*. *agalactiae* treatment decisions on farm level. Cure rates reported here can be useful to develop decision making economic models that will result in prevention of unnecessary exposure of cows to antimicrobials and maximize efficiency of *S*. *agalactiae* eradication programs.

## Conclusions

Results indicate that treatment with CLOXIMM or CEFIMM results in greater bacteriological cure rates, as compared with CEFIM or CONTROL. The low bacteriological cure of CEFIM observed in the present study does not justify its use in *S*. *agalactiae* eradication programs. Treatment with CLOXIMM was considered non-inferior to CEFIMM when non-inferiority margins of 0.20 or 0.25 were considered. Determination of non-inferiority was inconclusive for margins of 0.10 or 0.15. Thus, it should be pondered by veterinarians whether an expected 12.1 (5.6–18.4) percentage points increase in cure rate would justify the use of a fourth-generation cephalosporin, as opposed to a traditional IMM drugs (cloxacillin and ampicillin) to treat *S*. *agalactiae* subclinical mastitis.
